# Identifying *Plasmodium* P36 and P52 antigens for co-administration with circumsporozoite protein to enhance vaccine efficacy

**DOI:** 10.21203/rs.3.rs-4909396/v1

**Published:** 2024-09-24

**Authors:** Naveen Yadav, Anya C. Kalata, Rebekah A. Reynolds, Andrew Raappana, D. Noah Sather, Sean C. Murphy

**Affiliations:** 1 Department of Laboratory Medicine and Pathology, University of Washington, Seattle, WA, United States of America; 2 Center for Emerging and Re-emerging Infectious Diseases, University of Washington, Seattle, WA, United States of America; 3 Department of Microbiology, University of Washington, Seattle, WA, United States of America; 4 Seattle Children’s Research Institute, Seattle, WA, United States of America

**Keywords:** Plasmodium yoelii, Radiation and genetically attenuated sporozoites, CD8^+^ T cells, CD4^+^ T cells, malaria vaccine, circumsporozoite protein (CSP), P36, P52

## Abstract

Vaccines targeting the complex pre-erythrocytic stage of *Plasmodium* parasites may benefit from inclusion of multiple antigens. However, discerning protective effects can be difficult because newer candidates may not be as protective as leading antigens like the circumsporozoite protein (CSP) in the conventional pre-clinical mouse model. We developed a modified mouse model challenge strategy that maximizes the contribution of T cells induced by novel candidate antigens at the sporozoite challenge time point and used this approach to test *Plasmodium* P36 and P52 vaccine candidates alone and in concert with non-protective doses of CSP. Co-administration of P36 and/or P52 with CSP achieved 80–100% sterile protection in mice, compared to only 7–30% protection for each individual antigen. P36 and P52 vaccination induced murine CD4^+^ and CD8^+^ T cell responses, but not antibody responses. This study adds P36 and P52 as promising vaccine antigens that may enhance protection achieved by CSP vaccination.

## Introduction

Malaria is a potentially deadly parasitic disease caused by *Plasmodium* species that are spread across half of the globe by female *Anopheles* mosquitoes. Malaria causes an estimated 249 million annual cases and 608,000 deaths globally [1]. The emergence of drug and pesticide resistance highlights the urgent need for a highly effective malaria vaccine. RTS,S is a first-generation subunit malaria vaccine that targets the *P. falciparum* circumsporozoite protein (CSP) and is approved for use in humans, but RTS,S shows lower than desired efficacy and durability [2]. The newer R21 vaccine targets CSP and achieves improved efficacy of ~70% [3–5], but this level of protection is still below WHO target threshold of 90% [https://www.who.int/publications/i/item/9789240057463]. Developing more efficacious vaccines may hinge on adding more protective antigens to boost the efficacy of CSP-containing vaccines. Although whole parasite vaccine (WPV) approaches have shown complete protection in pre-clinical and clinical trials [6–17], WPV are hampered by complex vaccine production requirements and a need for direct venous administration. Therefore, to develop a more easily manufactured and simply administered vaccine, it is necessary to identify and credential additional novel protective candidate malaria antigens.

Complete protection against malaria can be achieved by targeting the parasite’s pre-erythrocytic stage [6, 18]. Protective immune mechanisms at this stage include antibody responses that can neutralize the parasite and prevent hepatocyte infection, and CD8^+^ T cells that can kill infected hepatocytes to arrest *Plasmodium* growth during this pre-erythrocytic stage and confer sterile protection [17, 21, 22]. CD4^+^ T cells are also crucial for maintaining protective antibodies and CD8^+^ T cell responses [14, 17, 23, 24]. Therefore, selection of candidate antigens that utilize either or both arms of the immune system can more effectively protect the host. Early arresting radiation-attenuated sporozoites (RAS) and genetically attenuated parasites (GAPs) have achieved complete protection in pre-clinical and clinical models and rely on both humoral and cellular arms of immune system [6, 10, 16, 25–28]. Protective candidate antigens from the early pre-erythrocytic stage may be broadly protective across different *Plasmodium* species due to amino acid sequence conservation of immunogenic regions of candidate antigens [29, 30]. The selected candidate antigen’s expression timing, concentration, and criticality of each antigen in the parasite life cycle all affect whether swift reactivation and targeting by the host immune cells will lead to protection. Based on these criteria, we evaluated *P. yoelii* P36 and P52 proteins as potential vaccine antigens.

P36 and P52 proteins are required by sporozoites (spz) for invasion of hepatocytes and establishment of the parasitophorous vacuole where the pre-erythrocytic parasite resides [31]. Parasites deficient in P36 and/or P52 lose their ability to develop in hepatocytes [32–34]. Abundant and early expression of P36 and P52 by spz make them good potential targets for the immune system. However, targeting these proteins in the spz stage is complicated for the host immune system as they are exposed to the immune system only after spz begin to invade host cells [31]. After spz invades host cells, P36 and P52 may be processed and presented by host cells for T cell targeting. Because of their conserved functions and restricted exposure to the immune system, P52 and P36 are less variable [29, 35]. Here, we hypothesized that targeting P36 and P52 with antibodies would block invasion of hepatocytes. Furthermore, we hypothesized that after invasion of hepatocytes, P36, P52, and CSP could singly or together be targeted by host T cells, and targeting these proteins in an orchestrated manner could help neutralize the parasite. Therefore, we screened vaccines based on individual or combined antigens for protective efficacy.

A pre-clinical limitation for credentialing new protective candidate antigens is the traditional mouse malaria model [36, 37]. *Plasmodium* parasites that naturally infect mice (i.e., *P. yoelii, P. berghei*) complete their pre-erythrocytic stage in 2–2.5 days, as compared to 5–6 days for *P. falciparum* in humans. In mice, CD8^+^ T cells are critical to pre-erythrocytic vaccines but may not fully exert their protective effects in the short duration pre-erythrocytic stage as it takes more than two days to recruit such cells to the liver from draining lymphoid organs [6, 38]. This may be why some investigators have reported very high requirements for antigen-specific CD8^+^ T cells in mouse models of malaria [39]. Since both murine and human hosts have similar immune response kinetics, human T cells are much more activated, developed, and expanded during the 5–6 day human pre-erythrocytic *Plasmodium* infection as compared to the shorter infection in mouse models [6]. Humanized liver mice support *P. falciparum* infection and are being used to mimic the human liver stage of infection [40], however humanized mice with human liver cells are immunocompromised, which eliminates their use for vaccine studies. Instead, we designed an alternative two-dose (kinetic) challenge strategy with *P. yoelii* (Py) using conventional inbred BALB/cJ mice to better evaluate protective outcomes of candidate antigens. In this two-dose challenge strategy, we maximize the participation of candidate antigens specific T cells in liver against *Plasmodium* at challenge time point based on the T cells kinetics reported earlier by us and others [6, 41–44].

In this study, mice were vaccinated by subunit delivery of plasmid DNA encoding candidate antigen(s) by gene-gun (GG) to prime antigen-specific T cell responses in the host. Four weeks later, we reactivated the peripherally GG-primed antigen-specific T cells with a low dose (2×10^3^, 2K) of radiation-attenuated sporozoites (RAS) to initiate T cell recruitment to the liver. Four days after the RAS dose, at a time when T cells were expanded, we challenged mice with a high dose (1×10^4^, 10K) of wild-type (WT) Py spz to evaluate the protective potential of candidate antigens. We screened P36 and P52 antigens individually with this strategy and found them to be partially protective (~7% and 27% respectively). When P36 and/or P52 were mixed with a non-protective dose of CSP (20–30%), complete protection was reliably achieved (80–100%). Overall, this approach adds two much-needed antigens to the malaria vaccine pipeline and provides a path for identifying and credentialing candidate pre-erythrocytic antigens.

## Results

### Two-dose challenge strategy and screening of rationally selected candidate antigens P36 and P52

**Figure 1:**
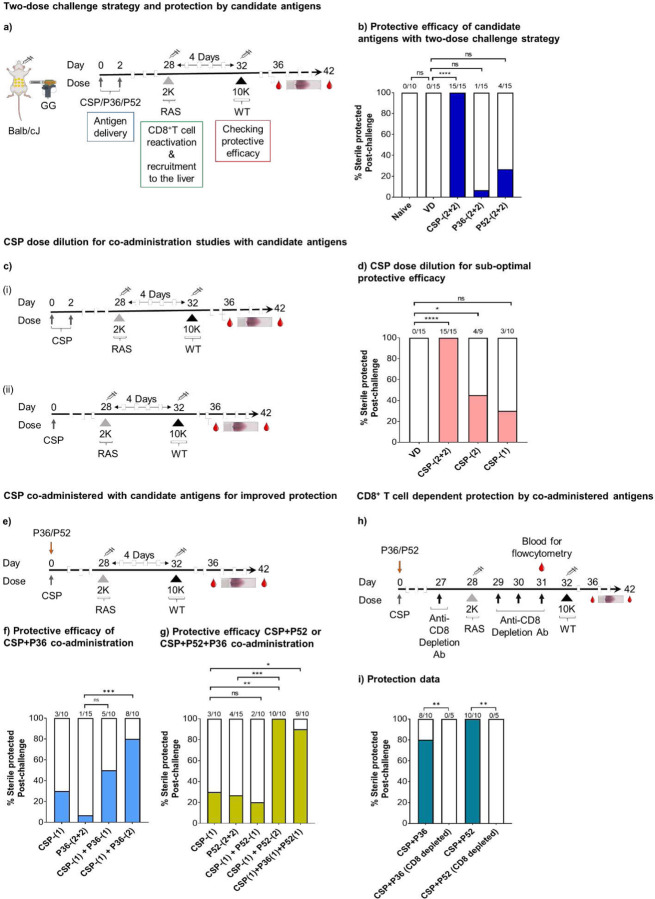
Two-dose challenge to test for protection by CSP, P36, and P52 antigens **a).** BALB/cJ mice were gene gun primed with DNA encoding the Py circumsporozoite protein (CSP)/P36 protein/P52 protein at days 0 and 2. On day 28, mice were given a dose of Py-RAS 2×10^3^ (2K) via the IV route. Finally, mice were subjected to a challenge dose of 1×10^4^ (10K) Py-WT on day 32 followed by blood smears to evaluate for sterile protection. Control group mice received empty vector DNA (VD) immunization via gene-gun, and/or a dose of Py-RAS and then were subjected to Py-WT challenge. **b).** Sterile protection outcomes for controls and CSP, P36, and P52 immunized groups. **c).** BALB/cJ mice were either (i) gene gun primed with DNA encoding the CSP protein at days 0 and 2 or (ii) gene gun primed with DNA encoding the CSP protein at day 0. On day 28, mice were given 2K Py-RAS via the IV route. Finally, mice were subjected to challenge with 10K Py-WT on day 32 followed by blood smears to evaluate for sterile protection. **d).** Sterile protection outcomes for control and Py-CSP-immunized groups. VD control group mice protection data acquired from panel b for comparison. **e).** For P36/P52 and CSP co-administration studies, mice were gene gun primed on day 0 with a single cartridge of CSP and one or two cartridges of P36/P52, received 2K Py-RAS on day 28, and 10K Py-WT challenge on day 32 followed by blood smears. Sterile protection outcomes for CSP and P36 (**f**), CSP and P52, and CSP, P52 and P36 studies (**g**) compared with CSP protection data from panel d, and P36 or P52 protection data from panel b. **h).** The role of CD8^+^ T cells in protection by two-dose challenge strategy was evaluated using T cell depletion strategy. BALB/cJ mice were gene gun primed 28 days earlier with CSP and P36 or CSP and P52 and were subjected to two-dose challenge. They were injected with CD8 depletion antibodies at indicated days followed by blood smears to evaluate for sterile protection. **i).** Protection data of CD8^+^ T cells depleted mice groups compared with protection data from panels F-G. N≤5 mice/group for panels a-g. Data compiled from two or more independent experiments. N=5 mice/group for panels h and i. Data were analyzed by Fisher Exact test: P<0.05 is considered significant. * P<0.05, ** P<0.01, *** P<0.001 **** P<0.0001. VD, vector DNA (control).

CD8^+^ T cell responses have important roles in achieving sterile protection at the *Plasmodium* pre-erythrocytic stage [17, 19–22]. Conventional pre-erythrocytic stage challenge strategies have identified relatively few protective candidate antigens such as CSP and TRAP (Thrombospondin-related anonymous protein). Some antigen screening efforts have found multiple immunogenic but non-protective targets [37, 45, 46]. It has been reported that protection in mouse models of malaria requires extremely high numbers of CD8^+^ T cells [39]. However, we recently showed that the short duration of the murine liver stage of infection could be a major reason for such a high threshold CD8^+^ T cell requirement against *Plasmodium* in the mouse model [6]. Based on the predicted CD8^+^ T cell kinetics against *Plasmodium* infection in longer duration liver stages as that seen in humans, we developed a modified mouse two-dose challenge strategy that makes use of expanded antigen specific CD8^+^ T cell to improve our ability to evaluate the contribution of candidate antigens in protection. Here, we primed the mice with desired candidate antigen(s) using two cartridges of plasmid DNA (0.5 µg plasmid DNA/cartridge) administered by gene-gun on days 0 and 2 to initiate peripheral CD8^+^ T cell responses. In some cases, administration consisted of fewer cartridges and/or dosing on only day 0 to reduce the immunogenicity of that antigen. Four weeks post-priming, we challenged those mice using a two-dose challenge strategy (Figure 1a). For the two-dose challenge used herein, mice first received a low dose of 2×10^3^ (2K) *P. yoelii*-radiation attenuated sporozoites (Py-RAS) to reactivate and recruit the peripherally-primed antigen specific CD8^+^ T cells to the liver. Four days after the Py-RAS dose, mice were challenged with a stringent high dose of 1×10^4^ (10K) *P. yoelii*-wild-type (Py-WT) spz. By this time, GG primed and Py-RAS reactivated antigen specific CD8^+^ T cell responses peaked in the liver [6]. The 10K wild-type challenge dose at this point is thus used to evaluate the protective efficacy of screening candidate antigen(s) specific CD8^+^ T cells that are present in the liver.

To assess the validity of this challenge strategy, we first immunized mice against the Py circumsporozoite protein (CSP) using a full gene gun dose (two cartridges per day on days 0 and 2 = ~2 µg CSP DNA). CSP is a dominant protective Py antigen in the BALB/cJ mouse model [47–49]. We found mice primed with this full CSP dose were all sterilely protected after two-dose challenge (Figure 1b). To determine if dose #1 (2K Py-RAS) of the two-dose challenge conferred any non-specific protection against challenge dose #2 (10K Py-WT), we tested protection by challenging naïve control mice that were gene gun immunized with control vector DNA (VD) alone followed by the two-dose challenge. All (100%) control mice developed blood stage infections (Figure 1b). Next, we screened two rationally selected, early pre-erythrocytic stage candidate antigens, P36 and P52. Initially, mice were primed using four cartridges of plasmid DNA in these studies. Both P36 and P52 were partially protective when delivered as single antigen vaccines. P36 sterilely protected 1 of 15 mice (<7% efficacy), and P52 protected 4 of 15 mice (27% efficacy) (Figure 1b). The observed low but partial protection encouraged further investigation into the ability of P36 and/or P52 to cooperate with CSP antigen specific responses to as multi-antigen vaccines.

### Co-administration of P36 and/or P52 with CSP enhances vaccine-induced protection

To evaluate P36 and P52 with CSP in multi-component antigens, we first needed to find an incompletely protective dose of CSP that could reliably highlight the added protective contribution of partner antigens. Dose de-escalation studies were performed to compare the standard dose of four cartridges (2 µg) of CSP plasmid DNA against fewer cartridges (i.e., less CSP). Two cartridges (1 µg) protected 45% of mice (Figure 1c.i, d) and one cartridge (0.5 µg) protected only 20–30% mice (Figure 1c.ii, d). Therefore, we used one cartridge of CSP DNA for immunization in subsequent multi-antigen vaccination studies. In these studies, we combined the single cartridge of CSP DNA with two cartridges of the partner antigen (either P36 and/or P52 encoding plasmid DNA) in initial multi-antigen studies described below; these gene gun vaccines were formulated with each antigen-coding plasmid on different aliquots of gold beads by highly parallel immunization to minimize antigenic competition. Mice were gene gun immunized with candidate antigen DNA followed by 2K Py-RAS dose after four weeks, and a 10K Py-WT challenge dose four days post Py-RAS (Figure 1e). Co-administration of a single cartridge of CSP with two cartridges of P36 on the same day sterilely protected 80% of mice (Figure 1f). Co-administration of a single cartridge of CSP with two cartridges of P52 on the same day sterilely protected all mice (100%) (Figure 1g). In contrast, co-administration of a single cartridge of each antigen (CSP+P36) and (CSP+P52) only partially protected mice (40% and 20%, respectively) (Figure 1f, g). However, when mice received a single cartridge of each antigen (CSP, P36 and P52), 80–100% were sterilely protected (Figure 1g). To ascertain the CD8^+^ T cell-dependent nature of protection in these immunized mice groups (CSP+P52 or CSP+P36) with this two-dose challenge strategy, we depleted CD8^+^ T cells in such mice after vaccination but just before challenging with 10K Py-WT (Figure 1h, S Figure 1). All CD8^+^ T cell-depleted mice became infected in both vaccination groups (Figure 1i), which highlights the requirement for CD8^+^ T cells in these vaccine regimens. The data thus suggests that three early pre-erythrocytic stage antigens, CSP, P36 and P52 can be combined in a synergistic manner to achieve sterile protection against malaria.

### CD4^+^ and CD8^+^ T cell responses against the candidate antigen proteins, P36 and P52

**Figure 2:**
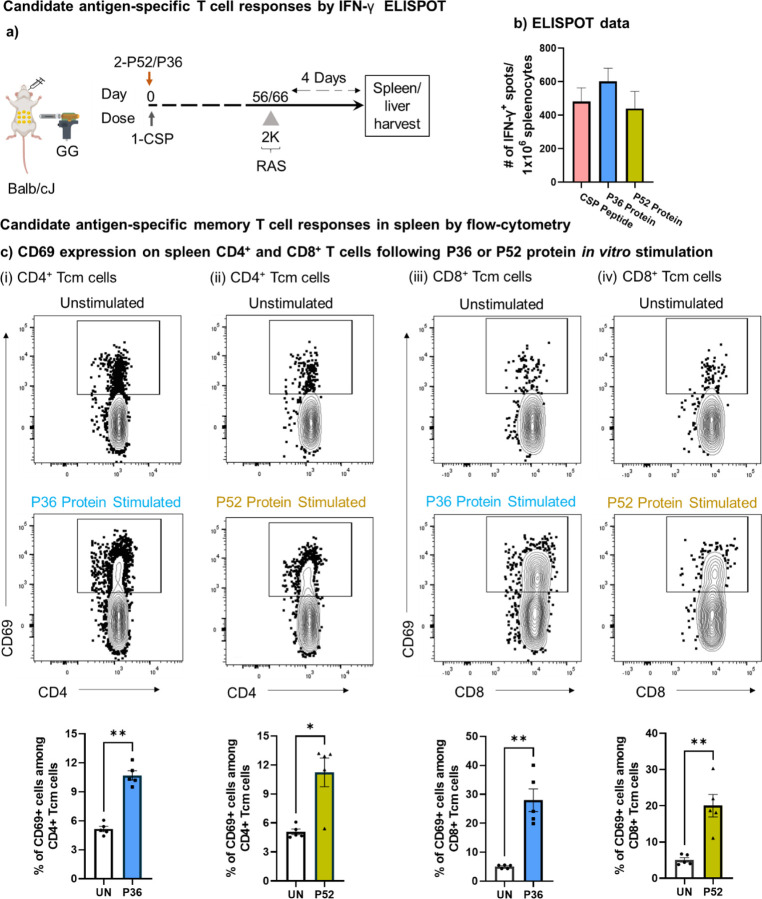
Antigen specific T cell responses against *P. yoelii* P36 and P52 antigens. **a).** Mice were immunized with the protective cocktail of DNA encoded CSP and P36/P52 and 8–9 weeks later received 2K Py-RAS followed by liver/spleen harvest for further cellular analysis by ELISPOT and flow-cytometry as shown. **b).** Splenocyte ELISPOT data for *in vitro* stimulation with CSP peptide, P52 protein, and P36 protein. **c).** Splenocytes from immunized mice groups depicted in panel A were stimulated overnight *in vitro* with the P36 (i and iii) or P52 (ii and iv) proteins and were tracked through flow cytometry to identify the activation of (i-ii) CD4^+^ and (iii-iv) CD8^+^ Tcm cells using the CD69 marker. A fluorescence minus one (FMO) control was used to gate on CD69 expressing CD8^+^ and CD4^+^ T cells (S figure 2, panel b). To quantify T cell activation following antigen stimulation, protein antigen was not added to unstimulated wells (UN). Frequency of T cell activation following either P36 or P52 protein stimulation was compared using the graphs at bottom of each panel using the marker CD69. N=5 mice per group. Data is representative of two independent experiments. Data are the mean ± SEM. Data were analyzed by Mann-Whitney test. *P*<0.05 is considered significant. * P<0.05, ** P<0.01.

**Figure 3:**
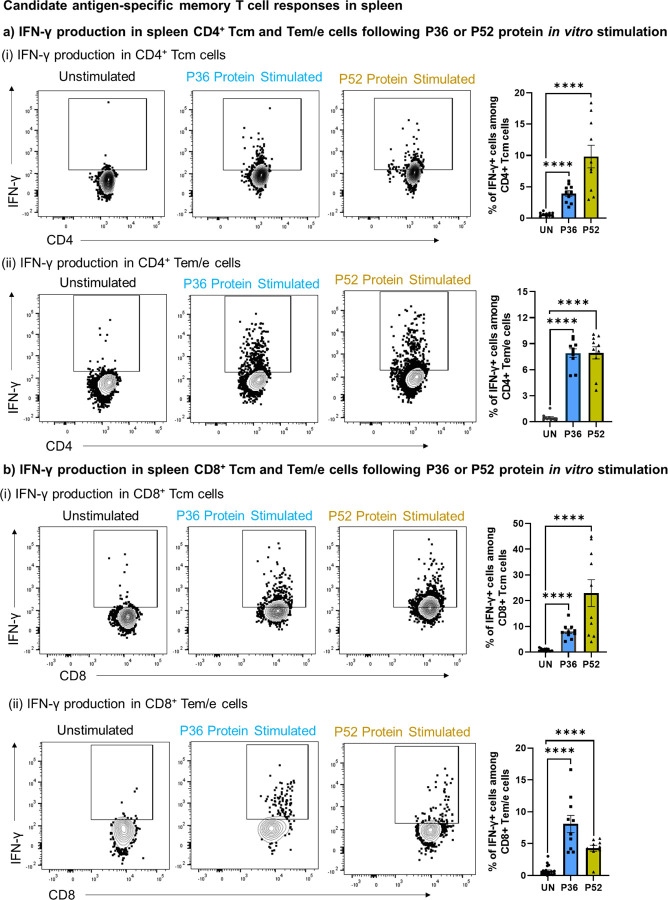

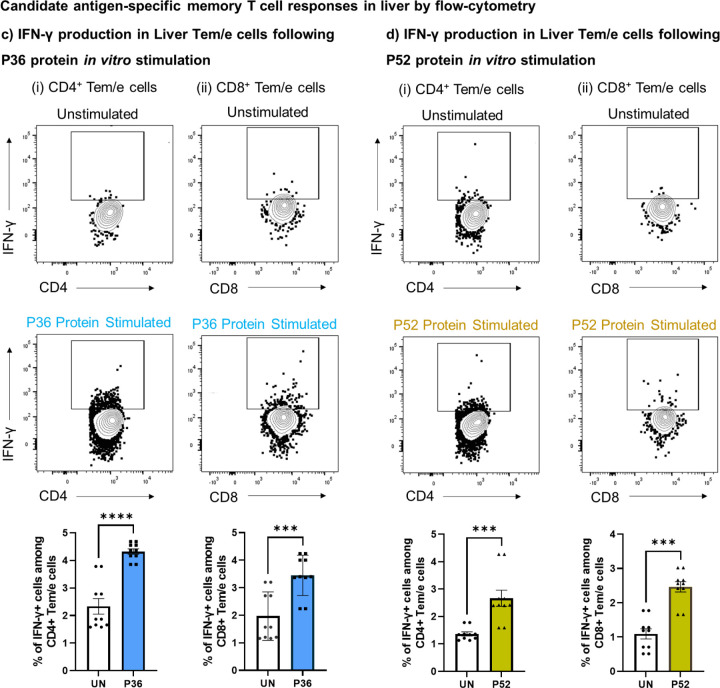
Memory T cell recall responses against *P. yoelii* candidate antigens, P36 and P52. Splenocytes and liver-mononuclear cells from immunized mice groups depicted in panel a of figure 2 were stimulated overnight *in vitro* with the P36 or P52 proteins and were tracked through flowcytometry to identify the activation of CD4^+^ and CD8^+^ memory T cells using the IFN-γ marker. **a-d).** Memory populations (Tcm and Tem/e) among CD4^+^ and CD8^+^ T cells of spleen (a and b) and liver (c and d) were tracked for IFN-γ production following P36 or P52 protein stimulation. A fluorescence minus one (FMO) control was used to gate on IFN-γ expressing CD8^+^ and CD4^+^ T cells (S figure 2, panel c and d). To quantify T cell activation following antigen stimulation, protein antigen was not added to unstimulated wells (UN). Frequency of T cell activation following either P36 or P52 protein stimulation was compared using the graphs on right side or at bottom of each panel using the marker IFN-γ. N=10 mice per group. Data is from two or three independent experiments. Data are the mean ± SEM. Data were analyzed by Mann-Whitney test. *P*<0.05 is considered significant. * P<0.05, ** P<0.01, *** P<0.001 **** P<0.0001.

Since T cells play a critical role in protection at the pre-erythrocytic stage, we further examined T cells in the spleen and liver at the time point corresponding to the wild-type challenge in this two-dose challenge strategy. Mice GG primed with the protective cocktail of either CSP+P52 DNA or CSP+P36 DNA were given 2K Py-RAS later to reactivate the P36- and P52-specific T cells. Four days after the Py-RAS dose, we harvested spleens and livers, and *in vitro* stimulated with P52 or P36 proteins to track the presence and activation of T cells by ELISPOT (Figure 2a-b). Further, using fluorescent markers we looked for the CD4^+^ and CD8^+^ T cells identity of those responding cells against the P36 and P52 proteins (Figure 2c, Figure 3).

IFN-γ ELISPOT demonstrated strong T cell responses against both the newly identified antigens P52 and P36, as well as against the co-administered CSP in spleen (Figure 2b). IFN-γ responses of both the candidate antigens, P36 and P52 were comparable to the dominant antigen CSP. Further using flow cytometry, we looked for the activation (CD69, IFN-γ) and identity of T cells (CD4^+^ and CD8^+^) responding to the P52 and P36 antigens. We found that upon *in vitro* stimulation with either antigens, P36 or P52, both CD4^+^ (Figure 2c.i, S Figure 2b.i) and CD8^+^ T cells (Figure 2c.ii, S Figure 2b.ii) significantly upregulated the surface expression of early activation marker, CD69. Further, we tracked the antigen-specific reactivation of CD4^+^ and CD8^+^ memory T cells by intracellular staining for IFN-γ production (Figure 3a-b, S Figure 2c-d). For that, we characterized CD4^+^ and CD8^+^ T cells into central memory (Tcm) and effector memory/effector (Tem/e) populations (S Figure 2a). We found that both memory populations in CD4^+^ and CD8^+^ T cells produced IFN-γ upon *in vitro* stimulation with P36 or P52 protein antigens (Figure 3a, b).

GG DNA immunization in mice is known to generate robust T cell responses against the target protein antigens in lymphoid organs [50]. However, for protection against pre-erythrocytic *Plasmodium* infection, T cells residency is required in the liver [50, 51]. Therefore, candidate antigens, P36 and P52 specific T cells likely need to be present in the liver to achieve high rates of protection. We found that liver-recruited P36 (Figure 3c) and P52 (Figure 3d) antigen-specific CD4^+^ and CD8^+^ Tem/e cells produced IFN-γ upon *in vitro* stimulation. Thus, the data suggested that DNA immunization induced the P36 and P52 antigen-specific CD4^+^ and CD8^+^ T cells in the host, and those cells could be reactivated by an encounter with the parasite (Py-RAS).

### Identification of candidate peptides using P36 and P52 peptide library

**Figure 4:**
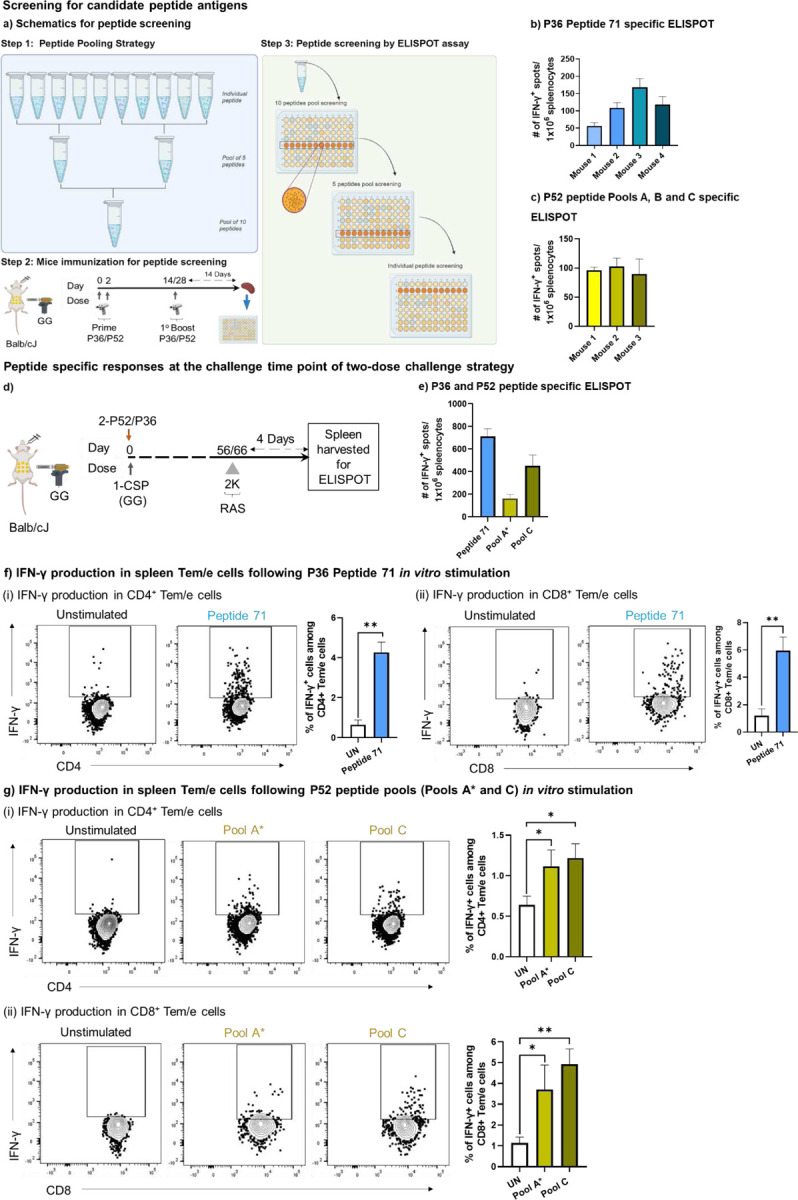
Identification and tracking of T cell responses against the P36 and P52 candidate peptides. **a).** Schematics of peptide pooling and screening strategy. For identifying the candidate immunogenic peptides from P36 or 52 peptide library, BALB/cJ mice were gene gun primed with DNA encoding P36 or P52 on days 0 and 2. Mice were later boosted again with P36 or P52 on day 14 or 28. Finally, mice were sacrificed 14 days post boosting and splenocytes were subjected to IFN-γ ELISPOT. **b).** Individual mice ELISPOT data for identified peptide from P36 peptide library (Peptide 71). **c).** Individual mice ELISPOT data compiled for three identified P52 peptide pools (Pool A*, Pool B, Pool C). **d).** BALB/cJ mice were gene gun primed with protective cocktails of DNA encoding both CSP plus either P36 or P52 at day 0. Mice were later received a reactivation dose of 2K RAS followed by spleen harvest for IFN-γ ELISPOT of Peptide 71 and Pool A*, Pool C. **e).** Number of IFN-γ positive spots per million splenocytes for P36 Peptide 71, P52 Pool A* and P52 Pool C. **f-g).** Splenocytes from the mice of panel D were *in vitro* stimulated either with P36 peptide 71 for CSP+P36 immunized group (f) or with P52 Pool A* and P52 Pool C for CSP+P52 immunized group (g) and Tem/e population among both CD4^+^ (i) and CD8^+^ T (ii) cells were tracked for IFN-γ production. For ELISPOT results are normalized against wells treated with control media. For flow cytometry fluorescence minus one (FMO) control was used to gate on the IFN-γ expressing CD8^+^ and CD4^+^ T cells (S Figure 2, panel d). To quantify T cell activation following antigen stimulation, peptide antigen(s) were not added to unstimulated wells. Frequency of T cells activation following either P36 or P52 antigen stimulation were compared with column graph on right side of each panel using the marker IFN-γ. N=3–4 mice per group for figures b-c. N=5 mice per group for panels d-g. Data are representative from two or three independent experiments. Data are the mean ± SEM. Data were analyzed by Mann-Whitney test. *P*<0.05 is considered significant. * P<0.05, ** P<0.01.

To identify T cell epitopes of P36 and P52 antigens, we screened overlapping peptide libraries for both proteins by IFN-γ ELISPOT. Mice were GG primed with DNA constructs as in Figure 4a. T cell responses were boosted by repeated GG immunization two to four weeks after priming. Two weeks after the final booster dose, draining lymphoid organs near the site of immunization (spleen and inguinal lymph nodes) were harvested, and cells were used to screen the peptide library by IFN-γ ELISPOT using pools of 10 peptides, then pools of five peptides, and finally individual peptides based on the ELISPOT results (Figure 4a). For P36, we identified one candidate peptide (Peptide 71) that responded robustly (VDRDILIYCNCSYNG) (Figure 4b). For P52, responses were distributed across three pools (Pool A, Pool B and Pool C) of 10 peptides each (Figure 4c). For P52 Pool A, we identified responses to two overlapping candidate peptides (IKHVMKMSFKKMTKK and MKMSFKKMTKKIKGC) and subsequently used these as a pool of two peptides (hereafter called “Pool A*”). For P52 (Pools B and C), we down-selected to 5 peptides per pool (Table S1). In further studies, we screened P36 Peptide 71 and P52 Pools A* and C for tracking peptide-specific responses as was previously done with full-length proteins (Figure 4d).

Splenocytes from the immunized mice were used for antigen-specific ELISPOT and flow cytometry (Figure 2A). IFN-γ ELISPOT demonstrated the presence of T cell responses against P36 Peptide 71 and against P52 Pools A* and C (Figure 4e). Further we screened those candidates using flow cytometry by tracking antigen-specific IFN-γ production from CD4^+^ and CD8^+^ Tem/e cells following *in vitro* antigen stimulation. For P36 Peptide 71, CD4^+^ (Figure 4f.i) and CD8^+^ Tem/e (Figure 4f.ii) cells responded. For P52 Pools A* and C, significant responses were also noted for both CD4^+^ (Figure 4g.i) and CD8^+^ Tem/e cells (Figure 4g.ii).

### Lack of measurable antibody responses against the candidate antigens

**Figure 5:**
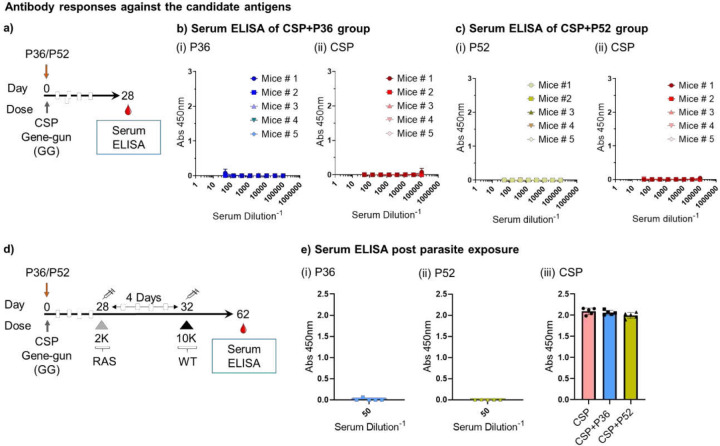
Lack of antibody responses to P52, P36, and repeatless CSP proteins. **a).** BALB/cJ mice were gene gun primed on day 0 with a single cartridge of repeatless CSP DNA and two cartridges of P36 or P52 DNA, and then bled for sera on day 28. **b).** IgG serum ELISA against (i) P36 and (ii) CSP of mice given CSP/P36. Positive controls shown in S Figure 3.A. **c).** IgG serum ELISA against (i) P52 and (ii) CSP of mice given CSP/P52. Positive controls shown in S Figure 3.b. **d).** BALB/cJ mice were primed on day 0 with a single cartridge of CSP DNA and two cartridges of P36 or P52 DNA, administered 2K Py-RAS on day 28, and challenged with 10K Py-WT on day 32 as shown followed by blood collection for sera on day 62. **e).** IgG serum ELISA for mice immunized as in panel d against (i) P36, (ii) P52, and (iii) CSP. Positive controls shown in S Figure 3.c. N=5 mice per group. Data is from an experiment. Data are the mean ± SD.

Antibodies also play an important protective role against the pre-erythrocytic stage infection [19, 20]. Candidate antigens P52 and P36 are highly expressed by the sporozoite stage of the parasite and can be targeted by antibodies preventing the invasion of the parasite into the hepatocyte [36]. Therefore, we looked for the presence of IgG responses against them after DNA immunization. Mice were GG immunized with protective cocktails of CSP+P36 or CSP+P52 plasmids (1 cartridge of CSP and 2 cartridges of P36 or P52) and after four weeks collected their blood for antibody measurement (Figure 5a). We screened them for total IgG responses against the repeatless CSP and candidate antigens (P36 and P52) (Figure 5b-c). There were no detectable IgG responses against P36 (Figure 5b.i) or P52 (Figure 5c.i). As expected, there were no detectable IgG responses against CSP in either group since the CSP antigen used herein to induce CD8^+^ T cell responses did not include the antibody-inducing repeat region (Figure 5b.ii, c.ii). All IgG positive controls were reactive against their cognate antigens (S Figure 3). However, it was recently reported that P36, P52, and CSP proteins from Py induce detectable antibody responses after full-length protein immunization [36]. Therefore, we also tested for antibody responses against P52, P36, and CSP in fully-protected CSP+P52 and CSP+P36 immunized mice four weeks after Py-WT spz challenge (Figure 5d). Antibody responses were still undetectable against P52 and P36 in these mice, whereas CSP-specific Ab responses were detected (Figure 5e). These data thus suggest that protection afforded by P36 and P52 is through T cell-based mechanisms.

### Kinetic expansion of RAS-induced CD4^+^ T cells

CD4^+^ T cells help in optimal expansion, functionality and maintenance of CD8^+^ T cells [52–55]. Hosts deficient in CD4^+^ T cells during vaccine priming or wild-type parasite encounter may not generate or appropriately rely on protective CD8^+^ T cell responses against *Plasmodium* [53, 56]. Earlier we showed that four days after RAS reactivation, there is an expanded pool of activated CD8^+^ T cells in the host [6]. To support this expanding pool of CD8^+^ T cells, CD4^+^ T cells are also expected to increase with similar kinetics [57, 58].

**Figure 6:**
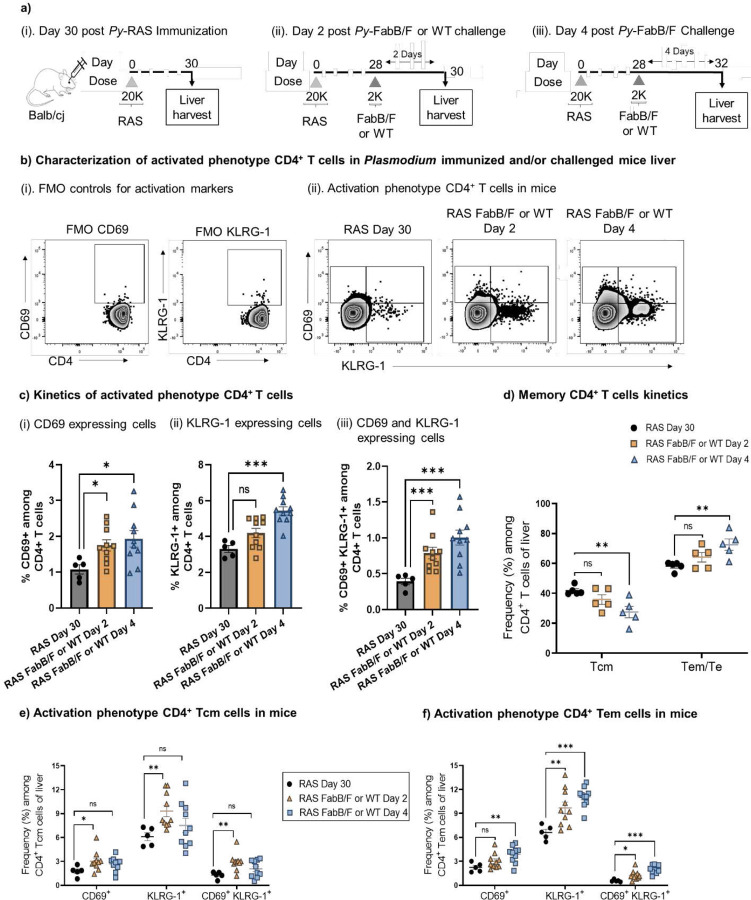
Kinetics of CD4^+^ T cell responses **a).** (i-iii) BALB/cJ mice were vaccinated 28 days earlier with 2×10^4^ (20K) *Py*-RAS and (ii-iii) were subjected to either WT or Fabbf challenge followed by liver harvest on days depicted in the figure to study the activated CD4^+^ T cell kinetics by flow cytometry. **b).** (i) Representative contour plots for CD69 and KLRG-1 FMO controls for positive gating of cells, (ii) representative contour plots for activated phenotype of CD4^+^ T cells (CD69^+^, KLRG-1^+^ and CD69^+^ KLRG-1^+^) in the liver of differently immunized mice as depicted in the panel A. **c).** Frequency (%) of (i) CD69^+^, (ii) KLRG-1^+^ and (iii) CD69^+^ KLRG-1^+^ cells among CD4^+^ T cells in livers of mice immunized as depicted in panel A. **d).** Frequency (%) of memory CD4^+^ T cells, central memory (Tcm) and effector memory (Tem) in Tm (CD44^Hi^) population. **e-f).** Frequency (%) of CD69^+^, KLRG-1^+^ and CD69^+^ KLRG-1^+^ cells among CD4^+^ Tcm (e) and Tem/e (f) population of the liver of differently immunized mice as depicted in the panel A. N=5 mice per group. Data are representative from one or two independent experiments. Data are the mean ± SEM. Data were analyzed by Mann-Whitney test. *P*<0.05 is considered significant. * P<0.05, ** P<0.01.

To track the kinetics of total CD4^+^ T cell expansion, we first immunized mice with 2×10^4^ Py-RAS (Figure 6a.i-iii). Four weeks later, we reactivated those cells using a 2K dose of genetically-attenuated (Py-FabB/f) or WT sporozoites as has been done previously for tracking CD8^+^ T cells expansion kinetics [6]. We then examined the reactivation and expansion kinetics of CD4^+^ T cells two (Figure 6a.ii) and four days later (Figure 6a.iii). There was continued activation of CD4^+^ T cells during days 2 and 4 (Figure 6b-f). Both CD69 single-positive, and CD69 and KLRG-1 double-positive cells were significantly increased in livers on days 2 and 4 (Figure 6c.i, iii). The mean percentage of KLRG-1 single-positive cells in the liver increased non-significantly on day 2 and significantly by day 4 (Figure 6c.ii). In memory T cell populations, Tcm phenotype cells decreased, whereas Tem/e phenotype cells increased over this period in the liver, likely corresponding with recruitment of Tcm cells out of the liver and recruitment of Tem/e cells into the liver (Figure 6d), consistent with classical T cell reactivation kinetics. Further, in liver, a higher percentage of both Tcm (Figure 6e; S Figure 4a) and Tem/e (Figure 6f; S Figure 4b) activated cells were present over time. Thus, we found alignment of CD4^+^ T cell activation and expansion alongside that of CD8^+^ T cells during this timeframe.

## Discussion

Clinical immunity against malaria can be acquired after years of frequent exposures to *Plasmodium* parasites [59], but sterile immunity is difficult to achieve with natural exposures [60]. However, vaccines can help achieve sterile immunity effectively in a short period [61]. Spz neutralizing Abs and liver infiltrating CD8^+^ T cells of diverse antigen specificity are critical for sterile protection. Screening and identifying protective antigens using pre-clinal mouse models is a major starting point for malaria vaccine development. Unfortunately, despite sterile protective capabilities of WPV approaches in mice, it has been difficult to define antigens beyond the likes of CSP and TRAP. Among the identified antigens, only CSP is currently used for approved human vaccination, but CSP vaccines currently achieve sub-optimal protective efficacy, which leads us to consider adding other conserved antigens to increase protection. A hurdle for new antigen discovery is the several days difference in the duration of the liver stage between *Plasmodium* parasites of rodents and those of humans (2–2.5 versus 5–6 days, respectively). In addition, MHC alleles in inbred mouse models are restricted compared to the outbred MHC diversity of humans. Therefore, we need a robust screening strategy in mouse models to prepare a portfolio of candidate antigens for further credentialing and down-selection.

To identify candidate antigens that can be added to CSP vaccines, we leveraged the Py-BALB/cJ mouse model and modified doses and schedules to reduce the protection achieved by the immunodominant Py-CSP protein. By adjusting the dosages and timing of antigens administered in a multi-antigen immunization regimen, we may be able to engineer an ideal vaccine-induced response that can help the host develop a wide pool of expanding T cells that can improve protective outcomes.

Identifying protective antigens is difficult because many antigens are immunogenic but ultimately non-protective in pre-clinical mouse models. Here, we used a two-dose challenge screening strategy to enhance our ability to detect antigens that enhance protection when combined with a CD8^+^ T cell-inducing CSP vaccine. The two-dose challenge strategy here differs somewhat from our previous report of two-dose challenge. In previous work, doses #1 and #2 were separated by 2–3 days and showed that CD8^+^ T cells increase during that time period [6]. In addition, we showed that single immunizations with low doses of RAS could protect against two-dose challenge [6]. Here, we extended the two-dose challenge interval to four days between doses #1 and #2 and increased the challenge dose 10-fold compared to our prior report. These changes were made to create a model whereby CSP-specific responses could participate in protection but CSP did not achieve complete protection alone. In doing so, we created the conditions whereby the protective effects of P36 and P52 as T cell antigens were more readily detected. The four-day interval between 2K Py-RAS and 10K Py-WT sporozoite challenge reported herein provided GG DNA-primed memory CD8^+^ T cells sufficient time to expand and recruit to the liver. Using this novel approach, we demonstrated that both P36 and P52 can be added to CSP T cell vaccines to achieve 80–100% sterile protection through both CD4^+^ and CD8^+^ T cell mechanisms. These findings provide a framework for building additional antigen screening approaches and for considering P36 and P52 as novel vaccine candidates for inclusion in multi-antigen malaria vaccines.

## Methods

### Mice

Female BALB/cJ mice (strain #000651; 4–6 weeks old) were obtained from Jackson Laboratories (Barr Harbor, ME), housed in an IACUC-approved animal facility at the University of Washington and used under an approved IACUC protocol 4317–01 (SCM). Infected mice were euthanized after detection of parasites in blood smears, while protected mice were euthanized after study completion. To euthanize mice, carbon dioxide inhalation was used as per approved IACUC guidelines at the University of Washington.

### Plasmodium parasites

Wild-type *P. yoelii* (*Py-*WT) 17XNL spz were harvested 14–18 days after an infectious blood meal by salivary gland dissection from infected *A. stephensi* mosquitoes reared at the Brotman Insectarium, University of Washington and Center for Mosquito Production and Malaria Infection Research (CeMPMIR) at the Center for Global Infectious Disease Research (CGIDR), Seattle Children’s Research Institute. Following dissection, sporozoite purification was conducted prior to irradiation using the Accudenz gradient method [62]. Briefly, after layering one-part salivary gland spz suspended in Schneider’s media over three parts 17% (w/v) Accudenz and centrifuging as reported, the top one-third of the gradient was transferred into 1.6 mL tubes and centrifuged at 13,300 x g for 4 minutes. Pellets from these tubes were combined, diluted with at least four parts of Schneider’s medium, and the spz were counted using a hemocytometer. Py*-*WT spz were radiation attenuated (Py-RAS) with 10,000 rads (Rad Source, Suwanee, GA).

### DNA cartridges

*Plasmodium yoelii* antigens P36 (PY01341), P52 (PY01340), and CSP (PY17X_0405400 without QGPGAP repeats) were cloned into vectors and then produced as vaccines. The antigen nucleotide sequences were codon optimized for mice (*Mus musculus*) and commercially synthesized (IDT). The antigens were then restriction-cloned into a vaccine vector (pUb.3) that contains an N-terminal ubiquitin tag. Plasmids were produced in *E. coli* HST08 and purified using an Endotoxin-free Maxiprep kit (Qiagen). Constructs were verified via Sanger Sequencing (Azenta Life Sciences). Vaccine plasmids plus 1:10 adjuvant plasmid (p7788 LT, encoding *Escherichia coli* heat-labile toxin) were loaded onto 0.8–1.5 µm gold beads (Technic Inc), and coated onto tubing to produce vaccine cartridges. Each cartridge contained approximately 500 ng of the vaccine plasmid and 50 ng of the adjuvant plasmid. The cartridges were quality controlled by verifying the DNA concentration. These vaccine cartridges were then used for particle-mediated epidermal delivery (PMED) using a PowderJect-style gene gun for needle-free delivery of plasmid DNA into trimmed abdominal skin.

### Vaccination and challenge

For sporozoite immunizations, Py-RAS was prepared by exposing Py-WT to 10,000 rads (Rad Source). For spz challenge, Py-WT were sequentially used four days after Py-RAS dosing. Immunization and challenge spz were administered intravenously (retro-orbital vein) in a volume of 100 μL per mouse. Dosages are indicated in separate experiments. For DNA vaccinations, plasmid DNA encoding the antigen of interest, was administered by gene gun either in a single shot or clustered on days 0 and 2.

### Blood smear endpoints

From day 4 post-Py-WT challenge, blood collected from mice by tail prick was smeared on glass slides, fixed with methanol, and Giemsa stained for detection of blood-stage *Plasmodium* parasites. Blood smears were monitored for the presence of parasites under an oil-immersion lens (1000X total magnification); blood collection was stopped either at day 10 post-challenge or when parasites were detected in blood smears. Animals were humanely euthanized upon detection of parasites in blood or at the end of the study with carbon dioxide inhalation.

### Quantification of antibody responses

IgG antibody responses against the protein antigens were quantitated by direct-immobilization ELISA as described previously [36].

### IFN-γ ELISPOT

For ELISPOTs, peptides or protein (1 μg/ml final) were combined with 1×10^6^ murine splenocytes and incubated for 18 h at 37°C as reported previously and developed following manufacturer guidelines. The numbers of activated T cells were calculated based on the spot-forming units counted in each well after deducting the spots count from media control wells.

### Protein or peptide antigen(s) stimulation and flow cytometry

Splenocytes were isolated and processed as previously described, and 0.7×10^6^ cells in triplicate were stimulated with 2 μg of antigen(s) for overnight. BFA was added 4 hours before processing cells for staining. Cells were blocked with normal mouse serum active as Fc block for 30 minutes. Surface staining was done by incubating cells for 20 minutes on ice with an antibody cocktail specific to cell surface markers (Table S2). Antibodies were diluted in staining buffer containing 50% Brilliant buffer by volume. Cells were then washed with staining buffer. Cells were then fixed and permeabilized using buffers used for intracellular staining as per the manufacturer guidelines. Intracellular staining was done by incubating cells for 30 minutes at room temperature in the dark. Cells were washed thrice with the permeabilization buffer, and then fixed with 1% formaldehyde reagent prepared in stain buffer. Stained cells were acquired on Fortessa instruments (BD Biosciences) and analyzed using FlowJo software (v10.10.0).

## Depletion of immune cells

Immune cell depletion studies were conducted using the antibodies as depicted in Table S3. Antibodies were diluted in PBS and administered intraperitoneally on days −1, +1, +2 and +3 relative to 2K Py-RAS dose of the two-dose challenge strategy.

## Statistics

Data are presented as the mean ± SEM. All comparisons of cell frequency were by the non-parametric Mann-Whitney tests or Fisher Exact test.

## Supplementary Material

Supplement 1

## Data Availability

All relevant data generated, analyzed and presented in this manuscript are available on request from the corresponding author.

## References

[1] Organization, W.H., World Malaria Report. 2023, World Health Organization: Geneva.

[2] Efficacy and safety of RTS,S/AS01 malaria vaccine with or without a booster dose in infants and children in Africa: final results of a phase 3, individually randomised, controlled trial. Lancet, 2015. 386(9988): p. 31–45. 25913272 10.1016/S0140-6736(15)60721-8 PMC5626001

[3] Datoo M.S., , Efficacy of a low-dose candidate malaria vaccine, R21 in adjuvant Matrix-M, with seasonal administration to children in Burkina Faso: a randomised controlled trial. Lancet, 2021. 397(10287): p. 1809–1818. 33964223 10.1016/S0140-6736(21)00943-0 PMC8121760

[4] Datoo M.S., , Efficacy and immunogenicity of R21/Matrix-M vaccine against clinical malaria after 2 years’ follow-up in children in Burkina Faso: a phase 1/2b randomised controlled trial. Lancet Infect Dis, 2022. 22(12): p. 1728–1736. 36087586 10.1016/S1473-3099(22)00442-X

[5] Datoo M.S., , Safety and efficacy of malaria vaccine candidate R21/Matrix-M in African children: a multicentre, double-blind, randomised, phase 3 trial. Lancet, 2024. 403(10426): p. 533–544. 38310910 10.1016/S0140-6736(23)02511-4 PMC7618965

[6] Yadav N., , More time to kill: A longer liver stage increases T cell-mediated protection against pre-erythrocytic malaria. iScience, 2023. 26(12): p. 108489. 38162031 10.1016/j.isci.2023.108489 PMC10755051

[7] Schofield L., , Gamma interferon, CD8+ T cells and antibodies required for immunity to malaria sporozoites. Nature, 1987. 330(6149): p. 664–6. 3120015 10.1038/330664a0

[8] Weiss W.R., , CD8+ T cells (cytotoxic/suppressors) are required for protection in mice immunized with malaria sporozoites. Proc Natl Acad Sci U S A, 1988. 85(2): p. 573–6. 2963334 10.1073/pnas.85.2.573 PMC279593

[9] Weiss W.R. and Jiang C.G., Protective CD8+ T lymphocytes in primates immunized with malaria sporozoites. PLoS One, 2012. 7(2): p. e31247. 22355349 10.1371/journal.pone.0031247 PMC3280278

[10] Yadav N., , Infectious sporozoite challenge modulates radiation attenuated sporozoite vaccine-induced memory CD8(+) T cells for better survival characteristics. Microbiol Immunol, 2022. 66(2): p. 41–51. 34674290 10.1111/1348-0421.12948

[11] Patel H., , Parasite load stemming from immunization route determines the duration of liver-stage immunity. Parasite Immunol, 2019. 41(7): p. e12622. 30854655 10.1111/pim.12622 PMC6584043

[12] Patel H., , *Frequent inoculations with radiation attenuated sporozoite is essential for inducing sterile protection that correlates with a threshold level* of Plasmodia liver-stage specific CD8(+) T cells. Cell Immunol, 2017. 317: p. 48–54. 28499490 10.1016/j.cellimm.2017.05.001

[13] Lyke K.E., , Attenuated PfSPZ Vaccine induces strain-transcending T cells and durable protection against heterologous controlled human malaria infection. Proc Natl Acad Sci U S A, 2017. 114(10): p. 2711–2716. 28223498 10.1073/pnas.1615324114 PMC5347610

[14] Roestenberg M., , Protection against a malaria challenge by sporozoite inoculation. N Engl J Med, 2009. 361(5): p. 468–77. 19641203 10.1056/NEJMoa0805832

[15] Roestenberg M., , Long-term protection against malaria after experimental sporozoite inoculation: an open-label follow-up study. Lancet, 2011. 377(9779): p. 1770–6. 21514658 10.1016/S0140-6736(11)60360-7

[16] Hoffman S.L., , Protection of humans against malaria by immunization with radiation-attenuated Plasmodium falciparum sporozoites. J Infect Dis, 2002. 185(8): p. 1155–64. 11930326 10.1086/339409

[17] Seder R.A., , Protection against malaria by intravenous immunization with a nonreplicating sporozoite vaccine. Science, 2013. 341(6152): p. 1359–65. 23929949 10.1126/science.1241800

[18] Fernandez-Ruiz D., , Liver-Resident Memory CD8(+) T Cells Form a Front-Line Defense against Malaria Liver-Stage Infection. Immunity, 2016. 45(4): p. 889–902. 27692609 10.1016/j.immuni.2016.08.011

[19] Cohen S., Mc G.I., and Carrington S., Gamma-globulin and acquired immunity to human malaria. Nature, 1961. 192: p. 733–7. 13880318 10.1038/192733a0

[20] Cohen S., Butcher G.A., and Crandall R.B., Action of malarial antibody in vitro. Nature, 1969. 223(5204): p. 368–71. 4980851 10.1038/223368a0

[21] Epstein J.E., , Live attenuated malaria vaccine designed to protect through hepatic CD8⁺ T cell immunity. Science, 2011. 334(6055): p. 475–80. 21903775 10.1126/science.1211548

[22] Ewer K.J., , Protective CD8+ T-cell immunity to human malaria induced by chimpanzee adenovirus-MVA immunisation. Nat Commun, 2013. 4: p. 2836. 24284865 10.1038/ncomms3836 PMC3868203

[23] Ishizuka A.S., , Protection against malaria at 1 year and immune correlates following PfSPZ vaccination. Nat Med, 2016. 22(6): p. 614–23. 27158907 10.1038/nm.4110 PMC11294733

[24] Mordmuller B., , Sterile protection against human malaria by chemoattenuated PfSPZ vaccine. Nature, 2017. 542(7642): p. 445–449. 28199305 10.1038/nature21060 PMC10906480

[25] Nussenzweig R.S., , Protective immunity produced by the injection of x-irradiated sporozoites of plasmodium berghei. Nature, 1967. 216(5111): p. 160–2. 6057225 10.1038/216160a0

[26] Dodoo D., , Measuring naturally acquired immune responses to candidate malaria vaccine antigens in Ghanaian adults. Malar J, 2011. 10: p. 168. 21689436 10.1186/1475-2875-10-168 PMC3132199

[27] Gwadz R.W., , Preliminary studies on vaccination of rhesus monkeys with irradiated sporozoites of Plasmodium knowlesi and characterization of surface antigens of these parasites. Bull World Health Organ, 1979. 57 Suppl 1(Suppl): p. 165–73. 120766 PMC2395714

[28] Clyde D.F., , Immunization of man against sporozite-induced falciparum malaria. Am J Med Sci, 1973. 266(3): p. 169–77. 4583408 10.1097/00000441-197309000-00002

[29] Ouattara A., , An In Silico Analysis of Malaria Pre-Erythrocytic-Stage Antigens Interpreting Worldwide Genetic Data to Suggest Vaccine Candidate Variants and Epitopes. Microorganisms, 2022. 10(6). 10.3390/microorganisms10061090 PMC9231253 35744609

[30] Tucker K.D., , Identification, Selection and Immune Assessment of Liver Stage CD8 T Cell Epitopes From Plasmodium falciparum. Front Immunol, 2021. 12: p. 684116. 34025684 10.3389/fimmu.2021.684116 PMC8138313

[31] Arredondo S.A., , The Micronemal Plasmodium Proteins P36 and P52 Act in Concert to Establish the Replication-Permissive Compartment Within Infected Hepatocytes. Front Cell Infect Microbiol, 2018. 8: p. 413. 30547015 10.3389/fcimb.2018.00413 PMC6280682

[32] VanBuskirk K.M., , Preerythrocytic, live-attenuated Plasmodium falciparum vaccine candidates by design. Proc Natl Acad Sci U S A, 2009. 106(31): p. 13004–9. 19625622 10.1073/pnas.0906387106 PMC2714279

[33] van Schaijk B.C., , Gene disruption of Plasmodium falciparum p52 results in attenuation of malaria liver stage development in cultured primary human hepatocytes. PLoS One, 2008. 3(10): p. e3549. 18958160 10.1371/journal.pone.0003549 PMC2568858

[34] Ishino T., Chinzei Y., and Yuda M., Two proteins with 6-cys motifs are required for malarial parasites to commit to infection of the hepatocyte. Mol Microbiol, 2005. 58(5): p. 1264–75. 16313615 10.1111/j.1365-2958.2005.04801.x

[35] Garzon-Ospina D., , Identifying Potential Plasmodium vivax Sporozoite Stage Vaccine Candidates: An Analysis of Genetic Diversity and Natural Selection. Front Genet, 2018. 9: p. 10. 29422913 10.3389/fgene.2018.00010 PMC5788960

[36] Vigdorovich V., , Coimmunization with Preerythrocytic Antigens alongside Circumsporozoite Protein Can Enhance Sterile Protection against Plasmodium Sporozoite Infection. Microbiol Spectr, 2023. 11(2): p. e0379122. 36847573 10.1128/spectrum.03791-22 PMC10100930

[37] Speake C., , Identification of Novel Pre-Erythrocytic Malaria Antigen Candidates for Combination Vaccines with Circumsporozoite Protein. PLoS One, 2016. 11(7): p. e0159449. 27434123 10.1371/journal.pone.0159449 PMC4951032

[38] Soulard V., , Plasmodium falciparum full life cycle and Plasmodium ovale liver stages in humanized mice. Nat Commun, 2015. 6: p. 7690. 26205537 10.1038/ncomms8690 PMC4525212

[39] Schmidt N.W., , Extreme CD8 T cell requirements for anti-malarial liver-stage immunity following immunization with radiation attenuated sporozoites. PLoS Pathog, 2010. 6(7): p. e1000998. 20657824 10.1371/journal.ppat.1000998 PMC2904779

[40] Minkah N.K., Schafer C., and Kappe S.H.I., Humanized Mouse Models for the Study of Human Malaria Parasite Biology, Pathogenesis, and Immunity. Front Immunol, 2018. 9: p. 807. 29725334 10.3389/fimmu.2018.00807 PMC5917005

[41] Whitmire J.K., Eam B., and Whitton J.L., Tentative T cells: memory cells are quick to respond, but slow to divide. PLoS Pathog, 2008. 4(4): p. e1000041. 18404208 10.1371/journal.ppat.1000041 PMC2275797

[42] Osborn J.F., , Central memory CD8+ T cells become CD69+ tissue-residents during viral skin infection independent of CD62L-mediated lymph node surveillance. PLoS Pathog, 2019. 15(3): p. e1007633. 30875408 10.1371/journal.ppat.1007633 PMC6420010

[43] Kohlmeier J.E., , The chemokine receptor CCR5 plays a key role in the early memory CD8+ T cell response to respiratory virus infections. Immunity, 2008. 29(1): p. 101–13. 18617426 10.1016/j.immuni.2008.05.011 PMC2519120

[44] Danahy D.B., , Polymicrobial sepsis impairs bystander recruitment of effector cells to infected skin despite optimal sensing and alarming function of skin resident memory CD8 T cells. PLoS Pathog, 2017. 13(9): p. e1006569. 28910403 10.1371/journal.ppat.1006569 PMC5599054

[45] Murphy S.C., , A T-cell response to a liver-stage Plasmodium antigen is not boosted by repeated sporozoite immunizations. Proc Natl Acad Sci U S A, 2013. 110(15): p. 6055–60. 23530242 10.1073/pnas.1303834110 PMC3625320

[46] Müller K., , Low immunogenicity of malaria pre-erythrocytic stages can be overcome by vaccination. EMBO Mol Med, 2021. 13(4): p. e13390. 33709544 10.15252/emmm.202013390 PMC8033512

[47] Li S., , Priming with recombinant influenza virus followed by administration of recombinant vaccinia virus induces CD8+ T-cell-mediated protective immunity against malaria. Proc Natl Acad Sci U S A, 1993. 90(11): p. 5214–8. 7685119 10.1073/pnas.90.11.5214 PMC46686

[48] Rodrigues E.G., , Single immunizing dose of recombinant adenovirus efficiently induces CD8+ T cell-mediated protective immunity against malaria. J Immunol, 1997. 158(3): p. 1268–74. 9013969

[49] Bruna-Romero O., , Complete, long-lasting protection against malaria of mice primed and boosted with two distinct viral vectors expressing the same plasmodial antigen. Proc Natl Acad Sci U S A, 2001. 98(20): p. 11491–6. 11553779 10.1073/pnas.191380898 PMC58757

[50] Olsen T.M., , Prime-and-trap malaria vaccination to generate protective CD8(+) liver-resident memory T cells. J Immunol, 2018. 201(7): p. 1984–1993. 30127085 10.4049/jimmunol.1800740

[51] Lefebvre M.N. and Harty J.T., You Shall Not Pass: Memory CD8 T Cells in Liver-Stage Malaria. Trends Parasitol, 2020. 36(2): p. 147–157. 31843536 10.1016/j.pt.2019.11.004 PMC6937381

[52] Weiss W.R., , The role of CD4+ T cells in immunity to malaria sporozoites. J Immunol, 1993. 151(5): p. 2690–8. 8103069

[53] Carvalho L.H., , IL-4-secreting CD4+ T cells are crucial to the development of CD8+ T-cell responses against malaria liver stages. Nat Med, 2002. 8(2): p. 166–70. 11821901 10.1038/nm0202-166

[54] Mandala W.L., , The role of different components of the immune system against Plasmodium falciparum malaria: Possible contribution towards malaria vaccine development. Mol Biochem Parasitol, 2021. 246: p. 111425. 34666102 10.1016/j.molbiopara.2021.111425 PMC8655617

[55] Tse S.W., Radtke A.J., and Zavala F., Induction and maintenance of protective CD8+ T cells against malaria liver stages: implications for vaccine development. Mem Inst Oswaldo Cruz, 2011. 106 Suppl 1(0 1): p. 172–8. 21881772 10.1590/s0074-02762011000900022 PMC4206913

[56] Overstreet M.G., , CD4+ T cells modulate expansion and survival but not functional properties of effector and memory CD8+ T cells induced by malaria sporozoites. PLoS One, 2011. 6(1): p. e15948. 21245909 10.1371/journal.pone.0015948 PMC3014941

[57] Butler M.O., , Ex vivo expansion of human CD8+ T cells using autologous CD4+ T cell help. PLoS One, 2012. 7(1): p. e30229. 22279573 10.1371/journal.pone.0030229 PMC3257268

[58] Kervevan J. and Chakrabarti L.A., Role of CD4+ T Cells in the Control of Viral Infections: Recent Advances and Open Questions. Int J Mol Sci, 2021. 22(2). 10.3390/ijms22020523 PMC7825705 33430234

[59] Doolan D.L., Dobaño C., and Baird J.K., Acquired immunity to malaria. Clin Microbiol Rev, 2009. 22(1): p. 13–36, Table of Contents. 19136431 10.1128/CMR.00025-08 PMC2620631

[60] Pohl K. and Cockburn I.A., Innate immunity to malaria: The good, the bad and the unknown. Front Immunol, 2022. 13: p. 914598. 36059493 10.3389/fimmu.2022.914598 PMC9437427

[61] Richie T.L., , Sporozoite immunization: innovative translational science to support the fight against malaria. Expert Rev Vaccines, 2023. 22(1): p. 964–1007. 37571809 10.1080/14760584.2023.2245890 PMC10949369

[62] Kennedy M., , A rapid and scalable density gradient purification method for Plasmodium sporozoites. Malaria Journal, 2012. 11(1): p. 1–10. 23244590 10.1186/1475-2875-11-421 PMC3543293

