# Supplementary material for: Identifying Plasmodium P36 and P52 antigens for co-administration with circumsporozoite protein to enhance vaccine efficacy
Source: Res Sq. 2024 Sep 24:rs.3.rs-4909396. Preprint. [Version 1] doi: 10.21203/rs.3.rs-4909396/v1 (PMC11469399; doi:10.21203/rs.3.rs-4909396/v1)
Supplement: Supplement 1 [file NIHPPRS4909396V1-supplement-1.pdf]

Supplementary Files

This is a list of supplementary files associated with this preprint. Click to download.

- SupplementaryInformationYadavetal.pdf
